# Weekend Effect and Mortality After Emergency Laparotomy: A Retrospective Cohort Study With Complimentary Meta‐Analysis

**DOI:** 10.1111/ans.70277

**Published:** 2025-08-01

**Authors:** Hashim Al‐Sarireh, Ahmad Al‐Sarireh, Shahin Hajibandeh, Shahab Hajibandeh

**Affiliations:** ^1^ University of Leeds Leeds UK; ^2^ University of Cambridge Cambridge UK; ^3^ Queen Elizabeth Hospital Birmingham UK; ^4^ Swansea University Swansea UK; ^5^ Morriston Hospital Swansea UK

**Keywords:** laparotomy, mortality, weekend

## Abstract

**Aims:**

To evaluate the prognostic significance of the weekend effect in patients undergoing emergency laparotomy.

**Methods:**

A STROCSS‐compliant retrospective cohort study (in three centres between January 2014 and January 2022) with complementary PRISMA‐compliant meta‐analysis (last search on 10 February 2025) was conducted. All adult patients undergoing non‐traumatic emergency laparotomy were considered eligible. Emergency laparotomy during weekends (Saturday, Sunday and public holidays) was the prognostic factor of interest, and emergency laparotomy during weekdays (Monday, Tuesday, Wednesday, Thursday and Friday) was the comparison. Thirty‐day mortality was the outcome.

**Results:**

The cohort study included 1952 patients and a search of electronic databases identified five retrospective cohort studies including 5374 patients. Consequently, 7326 patients (weekend group: 2035; weekdays group: 5291) were included for analyses. Both groups were comparable in terms of median age (67 years vs. 65, *p* = 0.194), being an octogenarian (17.9% vs. 17.9%, *p* = 0.970), male sex (41.9% vs. 45.7%, *p* = 0.153), ASA I status (4.5% vs. 6.7%, *p* = 0.080), ASA II (33.6% vs. 35.2%, *p* = 0.524), ASA III (46.6% vs. 41.6%, *p* = 0.060), ASA IV (14.7% vs. 15.2%, *p* = 0.764), ASA V (0.6% vs. 1.3%, *p* = 0.249), need for bowel resection (54.0% vs. 57.6%, *p* = 0.172) and peritoneal contamination (26.4% vs. 29.2%, *p* = 0.236). There was no difference in the risk of 30‐day mortality between the two groups (OR: 1.04, 95% CI 0.87–1.25, *p* = 0.650; *I* ^2^ = 0%). The GRADE certainty was high.

**Conclusions:**

Robust evidence with high certainty suggests that the weekend effect does not influence the risk of mortality after emergency laparotomy. This could be explained by the standardisation of perioperative care in patients undergoing emergency laparotomy.

## Graphical abstract

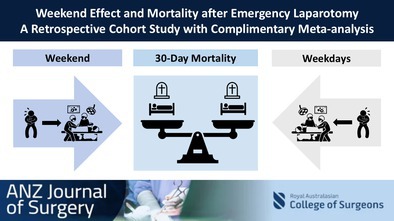

## 1 Introduction

The high risk of mortality associated with emergency laparotomy has encouraged exploring the predictors of mortality in this setting in order to provide a robust basis for perioperative decision making and multidisciplinary planning [1, 2]. The predictors of mortality after emergency laparotomy may include variables related to the physical status of the patients (age ≥ 80 [3], American Society of Anesthesiologists status [4], sarcopenia [4, 5], clinical frailty [5]), variables related to the socio‐economic status of the patients [6], variables related to the severity of the underlying abdominal pathology (peritoneal contamination [3], Hajibandeh index [2]) and variables related to the healthcare setting (surgeon's seniority [7], surgeon's subspeciality of interest [7], weekend effect [8], application of enhanced recovery after surgery [9]).

One potential healthcare setting‐related predictor of mortality after emergency laparotomy could be the ‘weekend effect’ which refers to variation in clinical outcomes in patients undergoing emergency laparotomy during weekends in comparison with weekdays. The weekend effect may be caused by differences in case mix or severity of disease in patients presenting during the weekend or by differences in staffing levels and reduced availability of resources leading to delays in diagnostics and procedures [10, 11, 12, 13, 14]. Hajibandeh et al. [8] demonstrated that the weekend effect in emergency General Surgery is variable across the world; although it seemed to be significant in the United States and Europe, it did not increase the risk of postoperative mortality in the United Kingdom [8]. Although the impact of the weekend effect on mortality in General Surgery has been evaluated previously [8], the prognostic significance of the weekend effect specifically in patients undergoing emergency laparotomy has not been established. In view of this, we aimed to perform a retrospective cohort study with complementary meta‐analysis to evaluate the prognostic significance of the weekend effect in patients undergoing emergency laparotomy.

## 2 Methods

### 2.1 Methodological and Reporting Compliance

The cohort study followed the Strengthening the Reporting of Cohort Studies in Surgery (STROCSS) guideline for observational studies [15], the methodology of the meta‐analysis followed the Cochrane Handbook for Systematic Reviews (version 6.4) [16]; the reporting of the meta‐analysis followed the Preferred Reporting Items for Systematic Reviews and Meta‐Analyses (PRISMA) 2020 statement standards [17].

### 2.2 Study Design and Patient Selection

#### 2.2.1 Cohort Study

The study was conducted in three centres in the United Kingdom (a Tertiary General Surgery centre and two District General Hospitals). The prospectively maintained hospital electronic medical record systems were used to identify all adult patients (age ≥ 18 years) who underwent emergency laparotomy between January 2014 and January 2022 due to non‐traumatic acute abdominal pathologies: intestinal perforation/obstruction/ischaemia/fistula, intra‐abdominal bleeding/collection, colitis or anastomotic leak. Emergency laparotomy due to trauma was an exclusion criterion.

#### 2.2.2 Meta‐Analysis

Two authors developed a search strategy using relevant keywords, operators, thesaurus headings and limits. The search was applied in MEDLINE, Scopus, CENTRAL, the ISRCTN registry, the ICTRP registry and ClinicalTrials.gov. The last date for the search was 10 February 2025, with no language restrictions. Moreover, the reference lists of relevant articles were explored to identify more eligible studies. All prospective and retrospective studies comparing postoperative mortality between patients who underwent non‐traumatic emergency laparotomy during weekends and weekdays were eligible for inclusion. Two independent authors screened the titles and abstracts of the identified articles and selected the eligible articles. The Quality In Prognosis Studies (QUIPS) tool [18] and the GRADE system [19] were used for the assessment of risk of bias and certainty, respectively. If there were any disagreements between the first two authors in any of the steps, a separate third author was consulted.

### 2.3 Prognostic Factor and Comparison

Emergency laparotomy during weekend (Saturday, Sunday and public holidays) was considered as the prognostic factor of interest and emergency laparotomy during weekdays (Monday, Tuesday, Wednesday, Thursday and Friday) was considered as the comparison of interest.

### 2.4 Outcome Measure

Thirty‐day mortality was the outcome of interest.

### 2.5 Data Collection

The following data items for each patient were collected: age, sex, ASA status, clinical frailty score, day of operation, indication for emergency laparotomy, peritoneal contamination, need for bowel resection and mortality outcomes. Data items were collected by two independent authors.

### 2.6 Data Synthesis and Statistical Analyses

#### 2.6.1 Cohort Study

The MedCalc software (version 23.1.6) was used for statistical analyses. The demographics, clinical characteristics and outcomes were summarised using median and interquartile range (IQR) for continuous variables and percentages for dichotomous variables. Continuous variables were compared using the Mann–Whitney *U* test and dichotomous variables using the chi‐square test. All statistical tests were two‐tailed, and statistical significance was assumed at *p* < 0.05.

#### 2.6.2 Meta‐Analysis

RevMan Web was used for comparison meta‐analysis. Random‐effects modelling was used to calculate odds ratio (OR) as summary effect measure. The results were presented in a forest plot with 95% confidence intervals (CIs). The unit of analysis was individual patient. *I* ^2^ was calculated using Cochran's *Q* test (*χ* ^2^) to quantify heterogeneity (low heterogeneity: *I* ^2^ 0%–25%; moderate heterogeneity: *I* ^2^ 25%–75%; high heterogeneity: *I* ^2^ 75%–100%). Separate analyses for studies with low overall risk of bias and leave‐one‐out analysis were performed as sensitivity analyses.

## 3 Results

**FIGURE 1:**
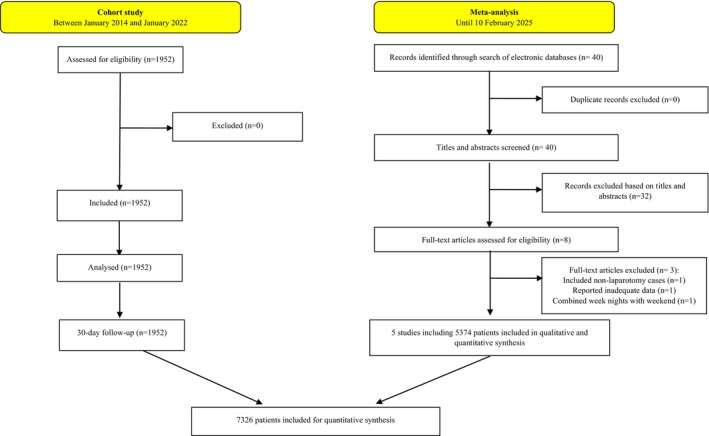
The study flow diagram.

The cohort study included 1952 patients and the search of electronic databases identified five retrospective [20, 21, 22, 23, 24] cohort studies including 5374 patients. Consequently, 7326 patients (weekend group: 2035; weekdays group: 5291) were included for analyses. Figure 1 demonstrates the study flow chart. Table 1 summarises the baseline characteristics of the included patients in the cohort study and Table 2 summarises the baseline characteristics of the included studies in the meta‐analysis.

**TABLE 1:** Baseline characteristics of the included patients in the cohort study.

	Weekend	Weekdays	*p* ^a^
No of patients	470	1482	
Age, median (IQR)	67 (51–76)	65 (51–76)	0.194
Age above 80, *n* (%)	84 (17.9%)	266 (17.9%)	0.970
Male, *n* (%)	197 (41.9%)	677 (45.7%)	0.153
Female, *n* (%)	273 (58.1%)	805 (54.3%)	0.153
ASA, *n* (%)			
I	21 (4.5%)	99 (6.7%)	0.080
II	158 (33.6%)	522 (35.2%)	0.524
III	219 (46.6%)	616 (41.6%)	0.060
IV	69 (14.7%)	226 (15.2%)	0.764
V	3 (0.6%)	19 (1.3%)	0.249
Clinical frailty scale, median (IQR)	2 (1–4)	2 (1–3)	0.097
Indication for laparotomy, *n* (%)			
Small bowel obstruction	194 (41.3%)	554 (37.4%)	0.130
Large bowel obstruction	69 (14.7%)	189 (12.8%)	0.282
Perforated peptic ulcer	25 (5.3%)	88 (5.9%)	0.617
Small bowel perforation	26 (5.5%)	62 (4.2%)	0.220
Colonic perforation	66 (14.0%)	261 (17.6%)	0.070
Intestinal ischaemia	29 (6.2%)	102 (6.9%)	0.591
Other	61 (13.0%)	226 (15.2%)	0.226
Need for bowel resection, *n* (%)	254 (54.0%)	854 (57.6%)	0.172
Peritoneal contamination, *n* (%)	124 (26.4%)	433 (29.2%)	0.236
30‐day mortality, *n* (%)	47 (10.0%)	160 (10.8%)	0.625

Abbreviations: ASA, American Society of Anesthesiologists; IQR, interquartile range.

^a^ Continuous variables were compared using the Mann–Whitney test and dichotomous variables were compared using the chi‐squared test.

**TABLE 2:** Baseline characteristics of the included studies in meta‐analysis.

First author, year, country	Journal	Design	Number of centres	Population	Definition of weekend	Definition of weekdays	Sample size			Weekend group vs. weekdays group	
							Total	Weekend group	Weekdays group	Age^a^	Male^b^
Current study, 2025, UK	—	Retrospective observational	3	Adult patients undergoing emergency laparotomy	Saturday or Sunday	Monday to Friday	1952	470	1482	67 (51–76) vs. 65 (51–76)	197/470 vs. 677/1482
Sylivris, 2023, New Zealand	*ANZ J Surg*	Retrospective observational	5	Adult patients undergoing emergency laparotomy	Saturday or Sunday	Monday to Friday	487	132	355	69 vs. 66	58/132 vs. 175/355
Patel, 2022, UK	*Cureus*	Retrospective observational	1	Adult patients undergoing emergency laparotomy	Saturday or Sunday	Monday to Friday	103	34	69	66 (18) vs. 67 (15)	21/34 vs. 25/69
Somasundram, 2020, UK	*Ann Med Surg*	Retrospective observational	1	Adult patients undergoing emergency laparotomy	Saturday or Sunday	Monday to Friday	263	92	171	58.1 (20.8) vs. 61.9 (20)	54/92 vs. 93/171
Butensky, 2020, USA	*Am Surg*	Retrospective observational	NR	Adult patients undergoing emergency laparotomy due to small bowel obstruction	Saturday or Sunday	Monday to Friday	2804	728	2076	67 (16.8) vs. 66 (17.3)	326/728 vs. 905/2804
Nageswaran, 2019, UK	*Ann R Coll Surg Engl*	Retrospective observational	4	Adult patients undergoing emergency laparotomy	Saturday or Sunday	Monday to Friday	1717	579	1138	68 (54–78) vs. 68 (54–78)	273/579 vs. 528/1138

Abbreviation: NR: not reported.

^a^ Mean (standard deviation) or median (interquartile range).

^b^ Number/total.

### 3.1 Risk of Bias in Included Studies

The risk of bias assessment using the QUIPS tool showed that all of the included studies were at low risk of bias (Table S1).

### 3.2 Baseline Patient Characteristics

The weekend group and weekdays group were comparable in terms of median age (67 years vs. 65, *p* = 0.194), being an octogenarian (17.9% vs. 17.9%, *p* = 0.97), male sex (41.9% vs. 45.7%, *p* = 0.153), ASA I status (4.5% vs. 6.7%, *p* = 0.080), ASA II status (33.6% vs. 35.2%, *p* = 0.524), ASA III status (46.6% vs. 41.6%, *p* = 0.060), ASA IV status (14.7% vs. 15.2%, *p* = 0.764), ASA V status (0.6% vs. 1.3%, *p* = 0.249), median clinical frailty scale (2 vs. 2, *p* = 0.097), need for bowel resection (54.0% vs. 57.6%, *p* = 0.172), peritoneal contamination (26.4% vs. 29.2%, *p* = 0.236) and indications for laparotomy (Table 1).

### 3.3 Thirty‐Day Mortality

#### 3.3.1 Cohort Study

**FIGURE 2:**
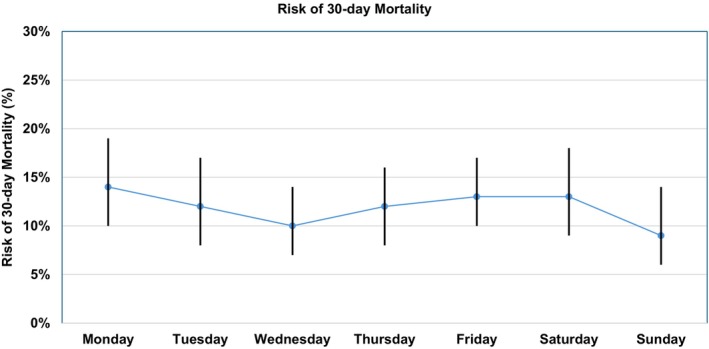
Risk of 30‐day mortality based on each day of week. The dots and vertical lines represent the risks of mortality on each day and the associated 95% confidence intervals, respectively.

The risk of 30‐day mortality was 10.0% (47 out of 470) in the weekend group and 10.8% (160 out of 1482) in the weekdays group. There was no difference in the risk of mortality between the two groups (*p* = 0.625). There was no difference in the risk of 30‐day mortality when each day of the week was analysed separately (Figure 2).

#### 3.3.2 Meta‐Analysis

**FIGURE 3:**
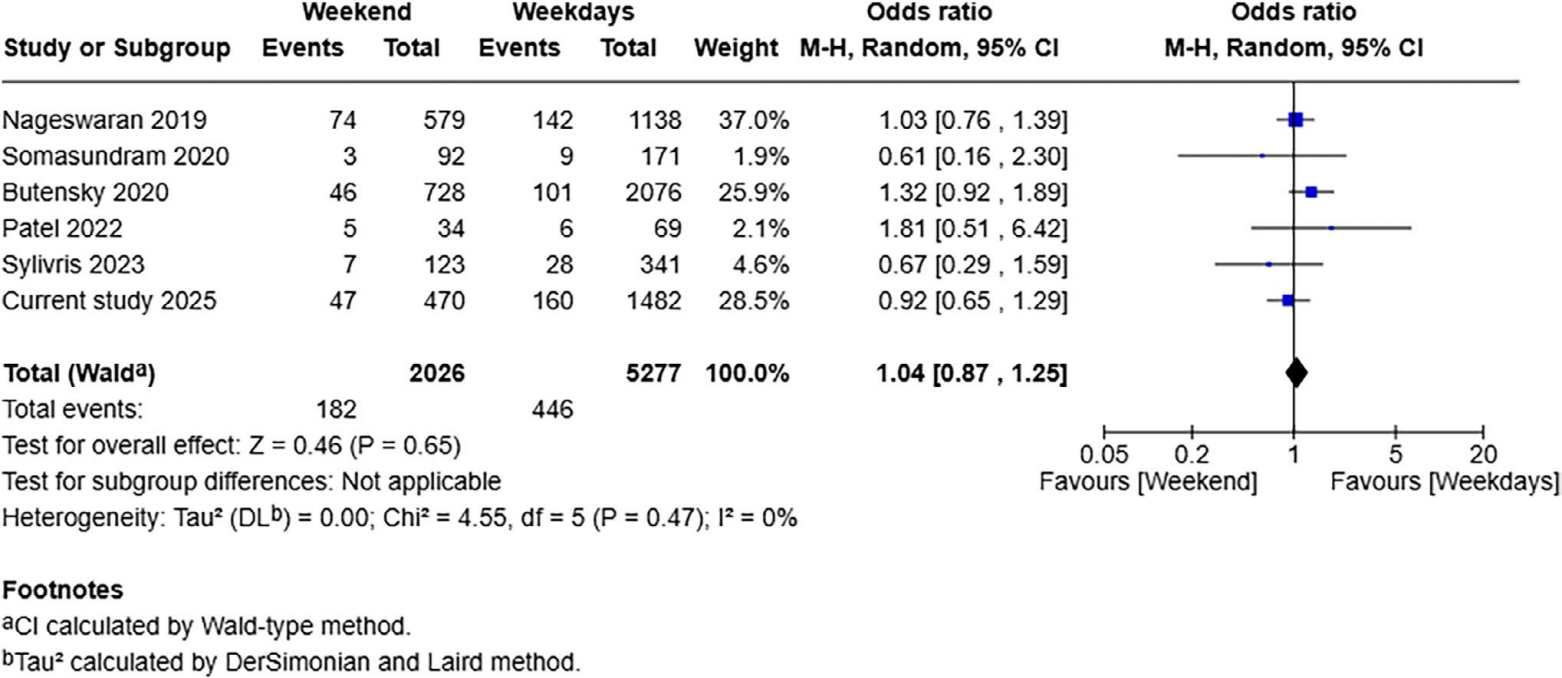
Forest plot for comparison of 30‐day mortality between the weekend group and weekdays group.

Analysis of 7303 patients from six studies (including the current cohort study) showed no difference in the risk of 30‐day mortality between the weekend group and weekday group (OR: 1.04, 95% CI 0.87–1.25, *p* = 0.650) (Figure 3). The between‐study statistical heterogeneity was low (*I* ^2^ = 0%, *p* = 0.470). The GRADE certainty was high. The direction of effect size did not change when studies with low risk of bias were analysed separately and when leave‐one‐out analysis was done.

## 4 Discussion

We completed a cohort study and complementary meta‐analysis to evaluate the impact of the weekend effect on postoperative mortality in patients undergoing emergency laparotomy. Analysis of 7326 patients (1952 patients from the cohort study and 5374 patients form literature) suggested that emergency laparotomy during the weekend does not increase the risk of postoperative mortality compared with weekdays. The between‐study heterogeneity was low and the GRADE certainty was high.

The findings of the current study have external validity. While there is no previous meta‐analysis on the impact of the weekend effect on mortality specifically after emergency laparotomy to compare our findings with, the prognostic significance of the weekend effect in general surgery has been evaluated. Hajibandeh et al. [8] conducted a meta‐analysis of 394 646 patients (10 studies) undergoing emergency general surgery operations and concluded that the weekend effect was variable across the world. Although it increased the risk of mortality in the United States and Europe, it did not increase the risk in the United Kingdom and South Africa [8]. Twahirwa et al. [25] conducted a retrospective study of 309 patients undergoing emergency laparotomy, which concluded that emergency laparotomies performed out of hours (nights and weekends) did not increase the risk of mortality [25]. Moreover, Ko et al. [26] and Elkbuli et al. [27] found no weekend effect in patients undergoing trauma laparotomy.

The comparable risks of mortality between laparotomies done during weekends and weekdays can be explained. Four of the included studies were from the United Kingdom in which perioperative management of patients who need emergency laparotomy follows the standards recommended by the National Emergency Laparotomy Audit (NELA) during weekdays and weekend [1]. This has improved access to specialist emergency surgical services, which resulted in improvement in operative mortality from 12.7% to 9.2% [1]. Similar standardisation of emergency care exists in Australia and New Zealand [28], which may explain the comparable mortality risks between the weekend and weekday groups in the study by Sylivris et al. [20].

The available evidence on the impact of the weekend effect on mortality after emergency laparotomy is robust enough to inform that the weekend effect should not be considered a predictor of mortality in this setting. This is supported by a large sample size in the current study, low clinical and statistical heterogeneity among the included studies, consistent findings through sensitivity analyses, and the high certainty (GRADE) of the available evidence. Nevertheless, some limitations should be taken into account. The retrospective design of the included studies would subject the results to selection bias. Moreover, the meta‐analysis included less than 10 studies; therefore, publication bias could not be assessed. Finally, four of the included studies were from the United Kingdom, which followed the same perioperative management. This may affect the generalisability of the findings; nevertheless, the findings of the other two studies that were from New Zealand and the United States were comparable with those from the United Kingdom. All of the included studies were conducted in countries with well‐organised health systems; therefore, the impact of the weekend effect on outcomes in countries with poorly organised health systems remains unanswered and should be the subject of interest in future studies.

## 5 Conclusions

Robust evidence with high certainty suggests that the weekend effect does not influence the risk of mortality after emergency laparotomy. This could be explained by standardisation of perioperative care in patients undergoing emergency laparotomy during weekdays and weekends across the world.

## Author Contributions

Conceptualisation: Shahab Hajibandeh. Methodology: Hashim Al‐Sarireh, Ahmad Al‐Sarireh and Shahab Hajibandeh. Data curation: Hashim Al‐Sarireh, Ahmad Al‐Sarireh and Shahab Hajibandeh. Validation and formal analysis: Hashim Al‐Sarireh, Ahmad Al‐Sarireh, Shahin Hajibandeh and Shahab Hajibandeh. Supervision: Shahab Hajibandeh. Visualisation: Shahin Hajibandeh and Shahab Hajibandeh. Project administration: Shahab Hajibandeh. Writing – original draft: Hashim Al‐Sarireh, Ahmad Al‐Sarireh, Shahin Hajibandeh and Shahab Hajibandeh. Writing – review and editing: Hashim Al‐Sarireh, Ahmad Al‐Sarireh, Shahin Hajibandeh and Shahab Hajibandeh.

## Ethics Statement

The authors have nothing to report.

## Consent

The authors have nothing to report.

## Conflicts of Interest

The authors declare no conflicts of interest.

## Supporting information


Table S1.

## Data Availability

The data that support the findings of this study are available from the corresponding author upon reasonable request.
