# Supplementary material for: Weekend Effect and Mortality After Emergency Laparotomy: A Retrospective Cohort Study With Complimentary Meta‐Analysis
Source: ANZ J Surg. 2025 Aug 1;95(10):2073–9. doi: 10.1111/ans.70277 (PMC12571939; doi:10.1111/ans.70277)
Supplement: Supplementary file 1 — Table S1. [file ANS-95-2073-s001.docx]

| **Supplementary Table 1. Results of risk of bias of the included case reports using the QUIPS (Quality In Prognosis Studies) tool** | | | | | | | |
| --- | --- | --- | --- | --- | --- | --- | --- |
| **First Author** | **Year** | **Study participation** | **Study attrition** | **Prognostic factor measurement** | **Outcome measurement** | **Study confounding** | **Statistical analysis** |
| Current study | 2025 | Low risk of bias | Low risk of bias | Low risk of bias | Low risk of bias | Low risk of bias | Low risk of bias |
| Sylivris | 2023 | Low risk of bias | Low risk of bias | Low risk of bias | Low risk of bias | Low risk of bias | Low risk of bias |
| Patel | 2022 | Low risk of bias | Low risk of bias | Low risk of bias | Low risk of bias | Low risk of bias | Low risk of bias |
| Somasundram | 2020 | Low risk of bias | Low risk of bias | Low risk of bias | Low risk of bias | Low risk of bias | Low risk of bias |
| Butensky | 2020 | Low risk of bias | Low risk of bias | Low risk of bias | Low risk of bias | Low risk of bias | Low risk of bias |
| Nageswaran | 2019 | Low risk of bias | Low risk of bias | Low risk of bias | Low risk of bias | Low risk of bias | Low risk of bias |
